# Coupled protein quality control during nonsense-mediated mRNA decay

**DOI:** 10.1242/jcs.261216

**Published:** 2023-05-23

**Authors:** Alison J. Inglis, Alina Guna, Ángel Gálvez-Merchán, Akshaye Pal, Theodore K. Esantsi, Heather R. Keys, Evgeni M. Frenkel, Robert Oania, Jonathan S. Weissman, Rebecca M. Voorhees

**Affiliations:** ^1^ Division of Biology and Biological Engineering, California Institute of Technology, 1200 E. California Blvd, Pasadena, CA 91125, USA; ^2^ Whitehead Institute for Biomedical Research, Massachusetts Institute of Technology, Cambridge, MA 02142, USA; ^3^ Howard Hughes Medical Institute, Massachusetts Institute of Technology, Cambridge, MA 02142, USA; ^4^ Department of Biology, Massachusetts Institute of Technology, Cambridge, MA 02142, USA; ^5^ David H. Koch Institute for Integrative Cancer Research, Massachusetts Institute Technology, Cambridge, MA 02142, USA

**Keywords:** mRNA, Nonsense-mediated decay, Quality control, Ubiquitin-proteasome pathway

## Abstract

Translation of mRNAs containing premature termination codons (PTCs) results in truncated protein products with deleterious effects. Nonsense-mediated decay (NMD) is a surveillance pathway responsible for detecting PTC containing transcripts. Although the molecular mechanisms governing mRNA degradation have been extensively studied, the fate of the nascent protein product remains largely uncharacterized. Here, we use a fluorescent reporter system in mammalian cells to reveal a selective degradation pathway specifically targeting the protein product of an NMD mRNA. We show that this process is post-translational and dependent on the ubiquitin proteasome system. To systematically uncover factors involved in NMD-linked protein quality control, we conducted genome-wide flow cytometry-based screens. Our screens recovered known NMD factors but suggested that protein degradation did not depend on the canonical ribosome-quality control (RQC) pathway. A subsequent arrayed screen demonstrated that protein and mRNA branches of NMD rely on a shared recognition event. Our results establish the existence of a targeted pathway for nascent protein degradation from PTC containing mRNAs, and provide a reference for the field to identify and characterize required factors.

## Teaser

**Summary:** Nonsense-mediated mRNA decay utilizes a coupled protein quality control pathway to coordinate degradation of both the aberrant mRNA and its associated nascent polypeptide chain.

## INTRODUCTION

Nonsense-mediated mRNA decay (NMD) is a broadly conserved and essential surveillance pathway that ensures the integrity of the transcriptome and regulates the levels of many cellular mRNA transcripts. NMD was initially identified for its role in recognizing and degrading aberrant, disease-causing mRNAs that contain a premature termination codon (PTC) within their open reading frame (Chang and Kan, 1979; Losson and Lacroute, 1979; Maquat et al., 1981). When translated, these mRNAs produce truncated proteins that can be aggregation-prone, develop gain of function phenotypes (Nonaka et al., 2009) or have dominant negative effects (Dietz et al., 1993; Hall and Thein, 1994; Kugler et al., 1995; Thein et al., 1990). NMD thus plays a critical role in maintaining cellular proteostasis by preventing expression of these potentially deleterious truncated proteins. Furthermore, one third of genetic disorders (Mort et al., 2008), including muscular dystrophy (Kerr et al., 2001) and cystic fibrosis (O'Sullivan, 2014) and many cancers (Anczuków et al., 2008; Karam et al., 2008; Perrin-Vidoz et al., 2002; Reddy et al., 1995; Ware et al., 2006) are the result of PTC-causing mutations that lead to recognition and degradation of the resulting mRNAs by NMD.

In addition to its role in transcriptome maintenance, NMD also regulates the levels of ∼10% of endogenous transcripts, facilitating rapid and flexible changes in gene expression in response to environmental and developmental stimuli (He et al., 2003; Lelivelt and Culbertson, 1999; Rehwinkel et al., 2005). NMD thus plays a fundamental role in diverse, but physiologically essential processes, including regulating the temporal expression of proteins during the cell cycle (Choe et al., 2014), degrading PTC-containing transcripts produced by somatic recombination during immune system development (Bruce and Wilkinson, 2003) and suppressing viral gene expression as a component of the innate immune response (Balistreri et al., 2014; Ramage et al., 2015).

Although there are no definitive rules as to what defines an NMD substrate, the composition of protein factors that decorate the 3′ UTR of an mRNA seem to either promote or prevent its degradation via NMD (Behm-Ansmant et al., 2007; Singh et al., 2008). For example, the positioning of poly(A)-binding protein (PABP) adjacent to the termination codon has been shown to be protective (Silva et al., 2008), whereas unusual physical features, such as upstream open reading frames (uORFs) and long 3′ UTRs are established cues for degradation by NMD (Behm-Ansmant et al., 2007; Mendell et al., 2004; Singh et al., 2008). It has also been observed that the many apparently ‘normal’ transcripts that are regulated by NMD have lower codon optimality and a higher rate of out-of-frame translation (Celik et al., 2017). However, the best characterized trigger for recognition by NMD is the presence of an intron downstream of a stop codon, which is commonly the result of genetic mutations or defects in alternative splicing (Shoemaker and Green, 2012). Splicing of these introns results in the deposition of an exon–junction complex (EJC) 24 nucleotides upstream of the splice site, which is retained upon packaging and export to the cytoplasm (Ballut et al., 2005; Hoskins and Moore, 2012; Le Hir et al., 2000; 2001). Because the majority of endogenous stop codons are localized within the last exon of protein coding genes, EJCs are typically removed during translational elongation (Dostie and Dreyfuss, 2002). The persistence of an EJC downstream of a stop codon is thus a characteristic of a PTC-containing mRNA and results in robust recognition by the NMD pathway (Gehring et al., 2003; Palacios et al., 2004).

Translation termination in the presence of a downstream EJC triggers NMD through a network of interactions between the core NMD factors UPF1, UPF2 and UPF3B; the downstream EJC; and the translational termination factors including eRF1 (also known as ETF1) and eRF3 (also known as GSPT1 and GSPT2) (Chamieh et al., 2008; Czaplinski et al., 1998; Kim et al., 2001; le Hir et al., 2001). Phosphorylation of UPF1 by SMG1 recruits a suite of RNA decay machinery to de-cap (DCP2) (Cho et al., 2009; Lai et al., 2012), deadenylate (CCR4-NOT) (Loh et al., 2013), cleave (SMG6) (Eberle et al., 2009; Huntzinger et al., 2008) and ultimately degrade the associated mRNA.

Like other mRNA surveillance pathways, NMD substrates are recognized and targeted for degradation co-translationally (Belgrader et al., 1993; J. Wang et al., 2002; Zhang and Maquat, 1997), resulting in the synthesis of a potentially aberrant nascent polypeptide chain. Pathways such as no-go and non-stop mRNA decay rely on a coordinated protein quality control pathway, known as ribosome-associated quality control (RQC) to both rescue the ribosome and concomitantly target the nascent protein for degradation (Doma and Parker, 2006; Frischmeyer et al., 2002; Juszkiewicz et al., 2018; van Hoof et al., 2002). In both cases, a terminally stalled ribosome or a collided di-ribosome triggers ribosome splitting (Becker et al., 2011; Pisareva et al., 2011; Shao et al., 2015; 2016; Shoemaker and Green, 2012) and nascent chain ubiquitylation by the E3 ligase LTN1 [facilitated by NEMF, TAE2, and P97 (also known as VCP)] (Brandman et al., 2012; Defenouillère et al., 2013; Lyumkis et al., 2014; Shao et al., 2013; 2015; Verma et al., 2013). The ubiquitylated nascent chain is then released from the ribosome by the endonuclease ANKZF1 (Vms1 in yeast) for degradation by the proteasome (Rendón et al., 2018; Verma et al., 2018).

Given the potential dominant-negative and proteotoxic effects of even small amounts of a truncated NMD substrate, it has been suggested that a similar protein quality control pathway might exist to recognize and degrade nascent proteins that result from translation of NMD mRNAs. Indeed, proteins produced from PTC-containing mRNAs are less stable than those from normal transcripts (Kuroha, Tatematsu, and Inada, 2009; Kuroha et al., 2013; Pradhan et al., 2021; Udy and Bradley, 2021). However, these observations are largely based on comparison of truncated products with longer, potentially more stable polypeptides, making it difficult to distinguish NMD-linked protein degradation from general cellular quality control mechanisms. Although recent work has directly tested this using a full-length protein product, there remains no defined mechanism of targeting and degradation, nor direct evidence for the involvement of the ubiquitin-proteasome pathway (Chu et al., 2021; Udy and Bradley, 2021). Furthermore, although it has been postulated that components of the RQC are involved in turnover of nascent NMD substrates (Arribere and Fire, 2018; Chu et al., 2021), the factors required for this process have not been systematically investigated. Because NMD is triggered at a stop codon, unlike no-go and non-stop decay, a putative NMD-coupled protein quality control pathway could require a fundamentally different strategy to initiate nascent protein degradation.

Here, we describe a reporter system that we have used to identify and interrogate a coupled protein quality control branch of NMD. We demonstrated that in addition to triggering mRNA degradation, NMD concomitantly coordinates degradation of the nascent polypeptide via the ubiquitin-proteasome pathway. Using this reporter system, we systematically identified factors required for NMD-coupled protein degradation, which are distinct from the canonical rescue factors of the RQC. Characterization of a coupled protein-degradation branch of NMD represents a new facet of our understanding of how the cell ensures the integrity and composition of its proteome, and sheds further light on the interplay between mRNA and protein quality control.

## RESULTS

### A reporter strategy to decouple mRNA and protein quality control in NMD

**Fig. 1.**
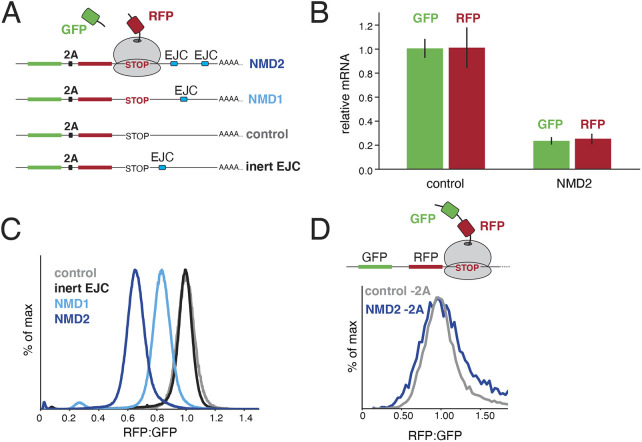
**Destabilization of nascent proteins from PTC-containing mRNAs.** (A) Schematic of the reporter strategy used to monitor protein and mRNA degradation in NMD. GFP and RFP are encoded in a single open reading frame separated by a viral 2A sequence. Positioning an intron within the 3′ UTR results in deposition of an exon junction complex (EJC) upon splicing, triggering NMD when compared to a matched control (control). Either one or two introns derived from the β-globin gene are inserted after the stop codon (NMD1 and NMD2, respectively). To control for the documented stimulation in translation that results from the presence of an EJC (Nott et al., 2004), we created a reporter in which the intron was positioned 12 nucleotides after the stop codon, a distance insufficient for recognition as an NMD substrate (inert EJC) (Nagy and Maquat, 1998). (B) T-Rex HEK293 cell lines stably expressing either the control or the NMD2 reporter were induced with doxycycline for 24 h and the total mRNA was then purified. Relative mRNA levels were determined by RT-qPCR using two sets of primers that anneal to the very 5′ region of the GFP and 3′ region of the RFP open reading frames respectively. The results were normalized to the control and the mean±s.d. from three independent experiments is displayed. (C) T-Rex HEK293 cell lines stably expressing the indicated reporters were analyzed by flow cytometry. The ratio of RFP:GFP fluorescence, normalized to the control reporter, is depicted as a histogram and quantified in Fig.S2E. (D) HEK293T cells were transiently transfected with versions of the control and NMD2 reporters in which the 2A sequence was scrambled, resulting in tethering of both GFP and RFP to the ribosome at the stop codon. Cells were analyzed by flow cytometry after 24 h and quantified in Fig. S2E.

To identify a putative NMD-linked protein quality control pathway, we developed a reporter system that uncouples mRNA and protein quality control during NMD. The reporter consists of a single open reading frame expressing GFP and RFP, separated by a viral 2A sequence that causes peptide skipping (Wang et al., 2015) (Fig. 1A; Fig. S1A). A robust example of an endogenous NMD substrate is the β-globin-encoding gene (*HBB*) with a nonsense mutation at codon 39, which results in a premature stop codon followed by an intron (Jing et al., 1998). We therefore reasoned that positioning the first intron of the human β-globin gene into the 3′ UTR of our reporter after the stop codon would also lead to its recognition as an NMD substrate, as has been previously reported (Chu et al., 2021; Lykke-Andersen et al., 2000; Pereverzev et al., 2015). We confirmed that the exogenous β-globin intron is efficiently spliced (Fig. S1B), and observed that the mRNA levels of the NMD reporter were ∼5-fold lower than a matched non-NMD control (Fig. 1B). We found that the GFP fluorescence of the NMD reporter and control correlated with their respective mRNA levels, as directly measured by quantitative (q)PCR, suggesting that GFP fluorescence can be used as a proxy for transcript levels (Fig. S1D). Furthermore, we saw that knockdown of the core NMD factor UPF1 specifically increased the GFP fluorescence of the NMD reporter (Fig. S1E–H) but had no effect on the matched control. We therefore concluded that our fluorescent reporter is recognized and degraded in an NMD-dependent manner. Finally, to ensure that these effects did not result solely from the increase in translation associated with the presence of an EJC (Nott et al., 2004), we also generated a reporter containing an EJC immediately following the stop codon, which is not recognized as an NMD substrate (inert EJC, Fig. 1A) (Nagy and Maquat, 1998). Indeed, the mRNA levels of this inert EJC construct were similar to those of our unspliced control (Fig. S1C).

After establishing that our reporters are subject to NMD-dependent mRNA degradation as expected, we sought to exploit them to determine whether there was an additional pathway dedicated to nascent protein degradation. For this, our reporter design has two important physical features. First, it can be used to deconvolute post-transcriptional versus post-translational effects on reporter levels. Upon translation, the GFP is released by the 2A sequence while the RFP remains tethered to the ribosome until the termination codon, where NMD is initiated by interaction between the downstream EJC and the ribosome. We reasoned that if there is an NMD-coupled pathway that triggers degradation of the nascent polypeptide, it would thus act only on the RFP but not the released GFP, resulting in a reduction in the RFP:GFP ratio in comparison to a matched control. In contrast, if NMD functions only in mRNA degradation, we would expect a decrease in both the RFP and GFP levels but would observe no change in the RFP:GFP ratio. Second, these reporters can specifically distinguish nascent protein degradation mediated by a coupled protein quality control pathway from non-specific recognition mediated by general cellular quality control machinery. Canonical NMD substrates contain PTCs that result in translation of a truncated protein, which might be misfolded and thus recognized and degraded by non-specific cytosolic quality control pathways (Popp and Maquat, 2013). By instead using an intact RFP moiety that is recognized as an NMD substrate only because of an intron in its 3′ UTR, any destabilization of RFP must result from a coordinated event that occurs prior to its release from the ribosome.

Indeed, using flow cytometry, we observed a decrease in RFP:GFP fluorescence for an NMD substrate compared to a matched control, in two different cell lines (Fig. 1C; Fig. S2A). Addition of a second β-globin intron to the 3′ UTR (Hoek et al., 2019) resulted in a larger decrease in both the mRNA levels and the RFP:GFP fluorescence ratio, suggesting the two effects may be tightly coordinated (Hoek et al., 2019). Although this decrease in RFP:GFP levels was consistent with NMD-dependent protein quality control, we sought to exclude several alternative models that could also account for this observation. First, we swapped the order of the RFP and GFP to rule out that differential maturation and/or turnover rates of the fluorophores could explain the decrease in the RFP:GFP ratio (Fig. S2B,C) (Amrani et al., 2004; Balleza et al., 2018). A similar effect was observed for this ‘reverse’ reporter, as previously reported (Chu et al., 2021). Second, we considered whether the decrease in RFP:GFP ratio could be the result of NMD-dependent deadenylation and 3′ to 5′ exonuclease degradation of the reporter mRNA (Chen and Shyu, 2003; Mitchell and Tollervey, 2003; Takahashi et al., 2003). However, we detected no difference in the relative mRNA levels of the RFP- and GFP-coding regions of the NMD substrate (Fig. 1B), confirming that the effect must occur post-transcriptionally.

Finally, we addressed two related possibilities – whether slow translational termination, which has been shown to occur on NMD substrates in yeast, although potentially not mammals (Amrani et al., 2004; Karousis et al., 2020), or SMG6-dependent endonucleolytic cleavage of the mRNA at the stop codon could explain the RFP:GFP ratio decrease (Eberle et al., 2009). The former could result in an increased dwell time of the ribosome at the stop codon when the ∼30 C-terminal residues of RFP remain occluded in the ribosomal exit tunnel and could potentially affect RFP folding and therefore fluorescence. The latter would lead to production of full-length GFP but truncated RFP and would be consistent with models proposed for putative NMD-coupled protein quality control in *C. elegans* (Arribere and Fire, 2018). However, appending a flexible linker to the C-terminus of RFP to ensure it is fully emerged from the ribosome at the stop codon did not affect the RFP:GFP ratio (Fig. S2D). This is consistent with the very long maturation time of RFP (in the order of minutes to hours; Balleza et al., 2018), which is therefore unlikely to be affected by any putative dwell time (in the order of milliseconds to seconds; Amrani et al., 2004) at the stop codon. Conversely, scrambling the 2A sequence, such that both the GFP and RFP are tethered to the ribosome at the stop codon, abolished the ratio difference (Fig. 1D; Fig. S2E). Together these data exclude that the NMD-dependent decrease in RFP:GFP ratio is due to changes in translation rate, processivity, peptide release, endonucleolytic cleavage or preferential 3′-5′ degradation.

### NMD-dependent protein degradation occurs via the ubiquitin-proteasome pathway

**Fig. 2.**
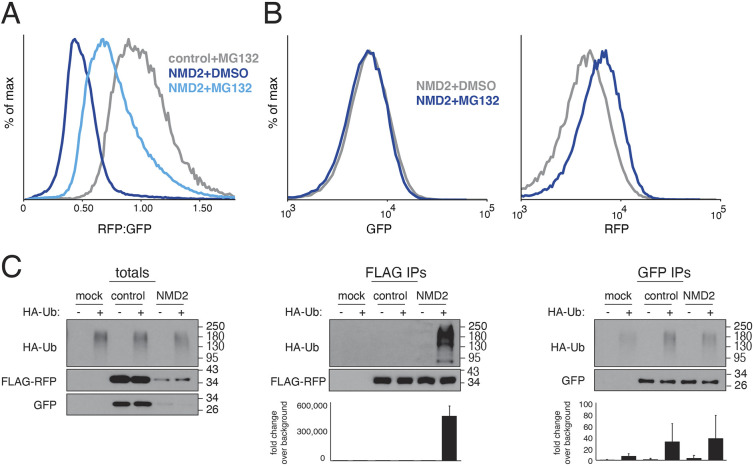
**NMD-dependent protein degradation occurs via the ubiquitin-proteasome pathway.** (A) Flow cytometry analysis of HEK293T cells transiently transfected with either the control or NMD2 reporter (Fig. 1A) and treated with the proteasome inhibitor MG132 or DMSO for 6 h. See quantification in Fig. S3A. (B) K562 CRISPRi cells stably expressing an inducible NMD2 reporter were treated with either MG132 or DMSO after induction of the reporter and analyzed by flow cytometry. Shown are the GFP (left) and RFP (right) channels for the indicated conditions displayed as a histogram, with fold change quantified in Fig. S3B. (C) HEK293T cells, stably expressing an HA-tagged ubiquitin (HA-Ub) were transiently transfected with either the control or NMD2 reporter (modified to incorporate a 3×FLAG tag at the N-terminus of RFP). To stabilize ubiquitylated species, cells were treated with MG132 prior to lysis. RFP was immunoprecipitated with anti-FLAG resin and GFP was purified using a GFP nanobody coupled to streptavidin resin (Pleiner et al., 2020). Ubiquitylated species were detected by western blotting for HA-Ub. The quantification of three independent replicates is shown below, with the mean±s.d. plotted.

Having established that an NMD-dependent decrease in RFP fluorescence occurs post-translationally, we tested whether inhibition of the ubiquitin-proteasome pathway could rescue the observed phenotype. We found that both the proteasome inhibitor MG132 and the E1 ubiquitin-activating enzyme inhibitor MLN7243 specifically increased the RFP:GFP ratio of the NMD reporter (Fig. 2A; Fig. S3A,C,D). Importantly, this increase was due to an effect on RFP and not GFP (Fig. 2B; Fig. S3B), consistent with the model that NMD-dependent protein degradation acts post-translationally and selectively toward the polypeptide associated with the ribosome at the PTC. To confirm that the observed changes in fluorescence reflect changes at the protein level, we directly tested for stabilization of RFP upon E1 enzyme inhibition by western blotting (Fig. S3D). The apparent absence of truncated RFP would be consistent with a model in which NMD-dependent protein quality control is initiated at the stop codon. Finally, we directly observed a marked increase in ubiquitylation of RFP, but not GFP, when expressed from our NMD reporter compared with a matched control, excluding potential indirect effects of ubiquitin-proteasome pathway inhibition (Fig. 2C). Therefore, we conclude that, in addition to its well-characterized role in mRNA degradation, NMD also triggers degradation of nascent proteins via the ubiquitin-proteasome pathway.

### Identification of factors required for NMD-coupled protein quality control

**Fig. 3.**
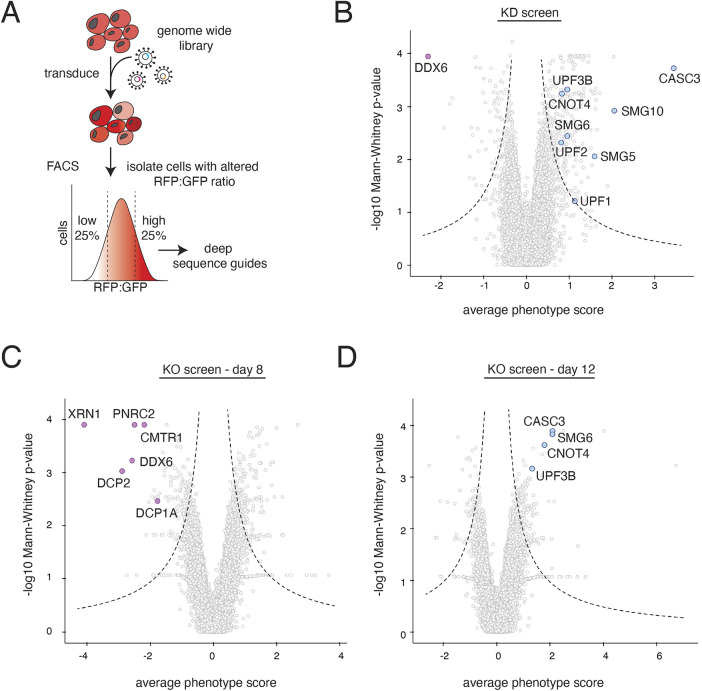
**Systematic characterization of factors required for NMD-coupled protein quality control.** (A) Schematic of the workflow used to carry out the FACS-based reporter screens to identify factors involved in NMD-linked nascent chain degradation. K562 reporter cell lines contained a tet-inducible NMD2 reporter and were infected with either a whole-genome CRISPRi sgRNA library or a CRISPR-KO library. Reporter expression was induced with doxycycline for 24 h prior to cell sorting. Cells were sorted based on ratiometric changes in RFP relative to GFP, and the sgRNAs expressed in those cells were identified using deep sequencing. The CRISPR knockout screen was sorted on days 8, 10 and 12 post-library infection to account for drop out of essential genes. The CRISPRi screen was sorted on day 8. (B) Volcano plot of the RFP:GFP stabilization phenotype (log2 for the three strongest sgRNAs per gene) and Mann–Whitney *P*-values from the genome-wide CRISPRi screen, with each point representing one gene. Genes falling outside the dashed lines are statistically significant. Each gray point represents a gene. Notable hits causing an increase in the RFP to GFP ratio are shown in light blue and include known NMD factors (UPF1, UPF2, UPF3B, SMG5, SMG6 and SMG10), the splicing factor CASC3 and the E3 ligase CNOT4. DDX6, a known suppressor of NMD, which causes a lower RFP to GFP ratio, is shown in purple. (C) Volcano plot as in B for the genome-wide CRISPR knock-out screen sorted at the day 8 timepoint. In purple are highlighted factors that cause a decrease in RFP relative to GFP. These include genes involved in mRNA de-capping (PNRC1, CMTR1, DCP1A and DCP2), DDX6 and the 5′-3′ exonuclease XRN1. (D) As in C but for day 12. In blue are shown known NMD factors (CASC3, SMG6 and UPF3B) and the E3 ligase CNOT4. Highlighted genes can be tracked across the 3 days of screening in Fig. S4A. The full datasets can be found in Tables S1 and S2.

Using our characterized NMD2 reporter, we systematically identified factors required for the protein degradation arm of NMD using a fluorescence-activated cell sorting (FACS)-based CRISPR interference (CRISPRi) (Horlbeck et al., 2016) and CRISPR knockout (CRISPR-KO) screen (Fig. 3A). We reasoned that the knockdown screen would enable study of essential proteins, including the core NMD factors UPF1 and UPF2 (Hart et al., 2017). Conversely, the knockout screen would identify factors that require near-complete depletion to induce a measurable phenotype, which can lead to false negatives in CRISPRi screens (Rosenbluh et al., 2017). To do this, we engineered two K562 human cell lines that expressed an inducible NMD2 reporter either alone or with the CRISPRi silencing machinery (Gilbert et al., 2014). We transduced the CRISPRi cell line with a single guide RNA (sgRNA) library targeting all known protein-coding open reading frames as previously described (hCRISPRi-v2) (Horlbeck et al., 2016). For the knockout screen, we used a novel 100,000 element library that targets all protein encoding genes (∼5 sgRNA/gene), which we used to simultaneously deliver both the genome wide sgRNA library and Cas9.

We hypothesized that depletion of factors required for NMD-coupled protein quality control would stabilize RFP, thereby increasing the RFP:GFP ratio. However, depletion of factors that impede NMD-coupled protein quality control would further decrease the RFP:GFP ratio. For the CRISPRi screen, after 8 days of knockdown, we sorted cells with high and low RFP:GFP ratios via FACS, and identified sgRNAs enriched in those cells by deep sequencing. For the knockout screen, we isolated cells with perturbed RFP:GFP ratios on days 8, 10 and 12 post infection of the CRISPR-KO library. We postulated that essential genes would be better represented at the earlier time points before their depletion becomes lethal, whereas factors that require complete depletion and/or have longer half-lives would be detected at later time points.

In both the knockdown and knockout screens, we find substantial differences between the hits identified here and those from earlier screens designed to identify factors primarily involved in NMD-dependent mRNA degradation (Alexandrov et al., 2017; Baird et al., 2018; Sun et al., 2011; Zinshteyn et al., 2021) suggesting that our reporter reflects a distinct aspect of the NMD pathway (Fig. 3B–D; Fig. S4A). However, we also identified several splicing and core NMD factors that affect the RFP:GFP ratio. For example, we found that the core component of the EJC, CASC3 (Gerbracht et al., 2020) is required for NMD-coupled protein degradation (Fig. 3B,D). Furthermore, depletion of several known NMD factors – UPF1, UPF2, UPF3B and SMG6 – increased the RFP:GFP ratio of our NMD reporter. Additionally, we also identified factors that appeared to enhance the degradation of RFP relative to GFP. On day 8 of the knockout screen, we found that several essential factors required for 5′ to 3′ mRNA degradation were enriched in the population of cells with lower RFP:RFP fluorescence (Fig. 3C). The phenotype scores for these essential factors decreased from day 8 to day 12, likely due to guide drop out, thereby validating the importance of examining the knock-out screen across multiple time points (Fig. S4A).

Together, these results suggest that there is a single, shared recognition step for both the mRNA and protein quality control branches of NMD, which requires recognition of an intact EJC downstream of the stop codon via interactions between the canonical NMD factors and the ribosome.

### NMD-coupled protein quality control is not mediated by canonical RQC factors

**Fig. 4.**
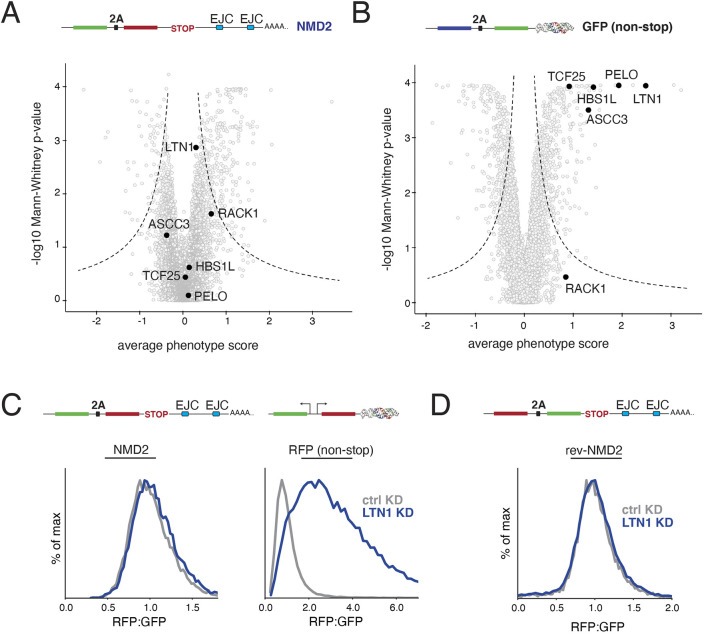
**NMD-linked protein degradation is not mediated by the canonical RQC pathway.** (A) Volcano plot of the NMD2 reporter CRISPRi screen as in Fig. 3A. Highlighted in black are factors involved in the canonical RQC (LTN1, RACK1, ASCC3, HBS1L, TCF25 and PELO). (B) For comparison, RQC factors are highlighted in black on a volcano plot for an earlier CRISPRi screen using a non-stop reporter (consisting of a BFP, a viral 2A skipping sequence and a GFP conjugated a triple helix moiety to stabilize the mRNA transcript, which would usually be degraded due to the lack of a stop codon) conducted using identical conditions as in A (Hickey et al., 2020). (C) K562 CRISPRi cells stably expressing either an inducible NMD2 reporter or a constitutively expressed non-stop reporter with matched GFP and RFP fluorophores (in this case, a functionally equivalent non-stop reporter with two separate promoters, one driving GFP, and the other RFP conjugated to the triple helix moiety; as in Hickey et al., 2020) were infected with a sgRNA targeting the E3 ligase LTN1. The RFP to GFP ratios for NMD2, and the GFP to RFP ratio for the non-stop reporter as determined by flow cytometry are displayed as a histogram and are quantified in Fig. S4B. (D) K562 CRISPRi cells expressing a reversed version of the NMD2 reporter (rev-NMD2) were infected with an sgRNA against LTN1 and analyzed as in C and are quantified in Fig. S4C.

Notably absent in both the knockdown and knockout screen were canonical components of the RQC pathway, suggesting that NMD substrates rely on an alternative strategy for nascent protein degradation. Because the CRISPRi screen was performed using the same platform and conditions as in earlier reporter screens for non-stop decay – including the same cell type, sgRNA library and sampling time point – the screens are directly comparable (Hickey et al., 2020). Although depletion of RQC factors including PELO and the E3 ubiquitin ligase LTN1 were identified in the non-stop reporter screen, neither are significant hits for NMD-dependent protein degradation in our system (Fig. 4A,B). We directly verified that LTN1 knockdown has no effect on our NMD reporter, or the ‘reverse’ reporter, but did have a marked effect on the fluorescence ratio of an established non-stop decay substrate (Fig. 4C,D; Fig. S4B,C). We therefore conclude that NMD-coupled protein degradation is mediated by a different set of factors.

### Factors required for NMD-coupled protein quality control

**Fig. 5.**
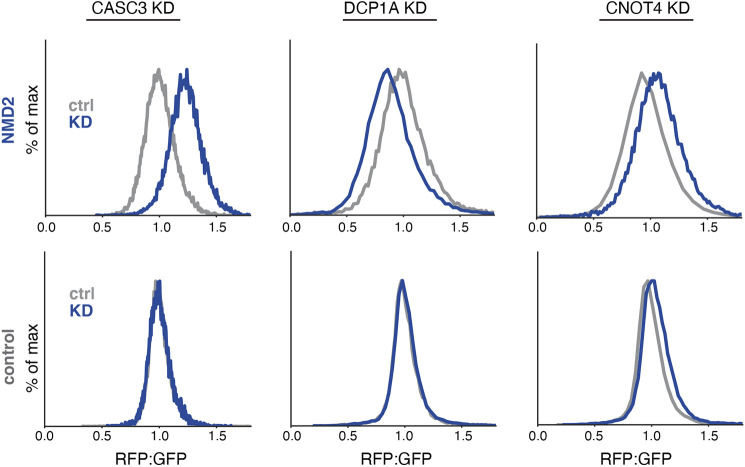
**Validation of factors involved in NMD-coupled protein quality control.** Factors of interest were individually depleted by sgRNA in K562 Zim3 CRISPRi cells expressing the indicated reporter. Displayed are the RFP:GFP ratios for the NMD2 (top) and control (bottom) reporters as determined by flow cytometry; see Fig. S5A,C,E for quantification. Similar results were obtained using reverse reporters as in Fig. S5B,D,F.

Hits from the FACS based reporter screens were validated using an arrayed screen with a matched control. These data confirmed that knockdown of CASC3 increased both the GFP levels and the RFP:GFP ratio of our NMD reporter (Fig. 5; Fig. S5A,C,E). The effect of CASC3 (also referred to as MLN51) depletion on our reporter is consistent with its established role as a splicing factor and a critical core component of the EJC (Le Hir et al., 2000; Andersen et al., 2006; Bono et al., 2006). Knockdown of the 5′ decapping enzyme DCP1A also increased GFP levels but decreased the RFP:GFP ratio. We confirmed these phenotypes were generalizable using our reverse GFP:RFP reporter (Fig. S5B,D,F).

Having observed that the nascent protein is directly ubiquitylated and degraded by the proteasome (Fig. 2), we were particularly interested in identifying an E3 ubiquitin ligase responsible for targeting the NMD-linked nascent chain for degradation. The core NMD factor UPF1 is an E3 ubiquitin RING ligase (Takahashi et al., 2008) and thus would be well-positioned to mediate nascent chain degradation during NMD. Previous studies have demonstrated that UPF1 stimulates proteasomal degradation of proteins expressed from NMD-targeted mRNA transcripts in yeast, with reporter stability significantly increased in *upf1* knockout strains; however, the mechanism underlying this phenotype is unclear and a direct role in nascent chain ubiquitylation by UPF1 was not shown (Kuroha et al., 2009). UPF1 was identified as a weak hit in our CRISPRi screen (Fig. 3B), and its depletion resulted in a shift in the RFP:GFP ratio of the NMD reporter (Fig. S1F–H). However, rescue of UPF1 knockdown with a RING mutant that disrupts binding with E2 ubiquitin-conjugating enzymes (Feng et al., 2017) phenocopied wild-type UPF1 in restoring both the GFP levels and RFP:GFP ratio of our NMD reporter (Fig. S6). This result would be inconsistent with a role for the RING domain of UPF1 in ubiquitylation of the nascent protein and suggests that the involvement of UPF1 might instead be upstream of the protein degradation branch.

In addition to UPF1, we identified four other E3 ubiquitin ligases in either the knockdown and knockout screen (KEAP1, MYLIP, CBLL1 and TRIM25). The RING ligase CNOT4 was the only hit to be identified in both screens; however, its effect was not specific to NMD substrates (Fig. 5; Fig. S5E–G), despite efficient depletion (Fig. S5G). It therefore is more likely playing a general role in cellular proteostasis, but is unlikely to be specifically involved in NMD-coupled nascent chain degradation.

**Fig. 6.**
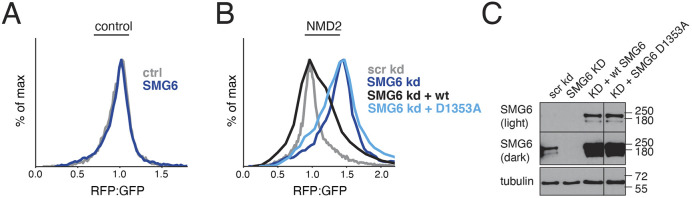
**NMD-coupled protein quality control is dependent on endonucleolytic cleavage of the mRNA by SMG6.** (A) HEK293T cells were treated with siRNA against SMG6 for 48 h, then were transiently transfected with the control reporter. The cells were analyzed by flow cytometry after 24 h (see quantification in Fig. S7A). (B) HEK293T cells were treated with siRNA against SMG6 for 48 h, then were transiently transfected with an siRNA-resistant version of either wild-type SMG6 or a PIN domain mutant (D1353A) along with the NMD2 reporter. The cells were analyzed by flow cytometry after 24 h (see quantification in Fig. S7B). Similar results were obtained with reverse reporters (Fig. S7D). (C) Levels of SMG6 in the samples from B were analyzed by western blotting against SMG6. Image representative of three repeats.

Additionally, the endonuclease SMG6 was also identified as a strong hit in both the knockdown and knockout screens (Fig. 3). Cleavage by SMG6 is considered a commitment step for degradation of NMD mRNAs, and we sought to determine whether the branchpoint of the protein and mRNA degradation pathways was upstream or downstream from this event. To do this we first used siRNA to deplete SMG6, and observed a considerable increase in the RFP:GFP ratio of our NMD reporter compared to its matched control (Fig. 6A,B; Fig. S7A,B). This phenotype could be rescued by ectopic expression of wild-type, but not a dominant-negative inactive mutant (Glavan et al., 2006), SMG6 for both our NMD and reverse reporters (Fig. 6B,C; Fig. S7B–D). Therefore, we conclude that the function of SMG6 is required for both mRNA and nascent-chain degradation in NMD, and in both cases depends on its endonuclease activity.

## DISCUSSION

Recognition of an NMD substrate occurs co-translationally, necessarily resulting in the production of a nascent, potentially cytotoxic, polypeptide chain. NMD typically reduces the mRNA level of its substrates 2–50-fold, depending on the transcript and function of the resulting protein product – a reduction that might not be sufficient to maintain proteostasis in the cell. As such, there has been consideration of whether NMD leverages an additional, post-translational pathway to directly target these nascent proteins for degradation (Chu et al., 2021; Kuroha et al., 2009; 2013; Pradhan et al., 2021; Udy and Bradley, 2021).

There are two plausible strategies by which protein degradation of NMD nascent chain can occur. Given that many NMD substrates are truncated and thus likely to misfold, they expose hydrophobic degrons that will be recognized by general cytosolic quality control machinery. However, this type of uncoordinated clearance strategy would risk the exposing the cell to transient dominant-negative or gain-of-function activity from these truncated or aberrant proteins. In contrast, a coordinated protein quality control pathway that co-translationally initiates protein degradation prior to dissociation from the ribosome would be more consistent with other mRNA surveillance pathways. Indeed, tight coupling of quality control to biogenesis is a strategy used throughout biology to ensure robust and efficient clearance of mRNA and protein products that fail during their maturation (Rodrigo-Brenni and Hegde, 2012).

In the case of NMD, the lack of a robust *in vitro* reconstitution system, the difficulty of deconvoluting post-transcriptional versus post-translational effects on expression of NMD substrates and the putative contribution of generalized quality control in turnover of the classical truncated NMD substrates has made it difficult to definitively identify this type of coordinated pathway. Using a fluorescent reporter strategy that addresses several of these technical challenges, we demonstrated that in mammals, NMD relies on a coupled protein quality control branch to concomitantly target the nascent protein for degradation via the ubiquitin proteasome pathway.

### A coupled protein quality control branch of NMD

**Fig. 7.**
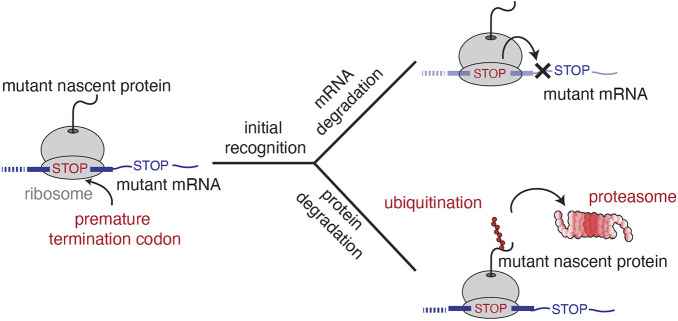
**Model for NMD-coupled protein quality control.** When the ribosome reaches the stop codon, NMD substrates are recognized in a context-dependent manner. These early recognition steps initiate two parallel pathways that rely on distinct suites of factors to concomitantly degrade the mRNA and nascent protein. We postulate that NMD-coupled quality control results in ubiquitylation of the nascent protein prior to its release from the ribosome where it subsequently degraded by the proteasome.

We propose the following working model for protein quality control during NMD in mammals (Fig. 7). As the ribosome reaches the stop codon during translational elongation, the protein composition of the downstream mRNA serves as the primary cue for initiating NMD. At this point, the nascent polypeptide remains tethered to the ribosome via the peptidyl tRNA. We postulate that the early recognition steps between the mRNA and protein quality control branches of NMD are shared, and rely on core NMD factors such as UPF1, UPF2, UPF3B and CASC3. NMD-coupled quality control is thus initiated through the canonical pathway for recognition of PTC-containing mRNAs that involves binding between the ribosome, NMD factors and the downstream EJC (Gerbracht et al., 2020; Chamieh et al., 2008; Czaplinski et al., 1998; Kim et al., 2001; le Hir et al., 2001). However, because our screens were designed to specifically query factors required for NMD-coupled protein quality control, we find substantial differences between hits identified here and those reported from earlier NMD RNA-degradation screens (Alexandrov et al., 2017; Baird et al., 2018; Sun et al., 2011; Zinshteyn et al., 2021). This discrepancy suggests that following recognition of an NMD substrate, the mRNA and protein quality control pathways diverge, relying on distinct sets of factors to target and degrade either the mRNA or nascent protein. However, the pathways are strictly linked, as evidenced by the requirement for endonucleolytic mRNA cleavage by SMG6 for efficient protein degradation.

We favor a model in which degradation of the nascent polypeptide is initiated prior to its release from the ribosome, as is common to other mRNA surveillance pathways and would minimize potential exposure of an aberrant protein to the cytosol. Consistent with this model we (1) found that only the nascent polypeptide tethered to the ribosome at the stop codon is subjected to NMD-coupled degradation (Figs 1D, 2B); and (2) we observe an NMD-specific destabilization of an intact, folded protein compared to a matched control. We therefore conclude that the nascent protein must be somehow ‘marked’ for degradation prior to its dissociation from the ribosome. However, our data is consistent with earlier studies that suggest that multiple rounds of translation are required before an mRNA is committed to NMD-dependent degradation (Hoek et al., 2019). We similarly observe incomplete degradation of the nascent chain (RFP), in line with only a proportion of ribosomes eliciting NMD-dependent ubiquitylation.

Following ubiquitylation of the nascent protein, it can then be safely released into the cytosol for degradation by the proteasome. In contrast to non-stop and no-go mRNA decay, where the primary cue for protein quality control is ribosome stalling (Brandman and Hegde, 2016), NMD is initiated at a stop codon and thus likely utilizes the typical strategy for nascent protein release and ribosome recycling. The manner by which the nascent protein is recognized as emanating from an NMD substrate is unclear – it has been suggested that, at least in yeast, termination at PTCs occurs more slowly than at a canonical stop codon, which could provide a kinetic window for ubiquitylation of the nascent protein (Amrani et al., 2004); however, no evidence for this has been found in human cells (Karousis et al., 2020). We therefore cannot differentiate whether nascent protein ubiquitylation occurs simultaneously or immediately following translational termination, but we favor a model where ubiquitylation is initiated prior to dissociation of the nascent chain from the ribosome.

### A potential role for the RQC pathway in NMD-coupled protein quality control

Several non-mutually exclusive models have been proposed for how to coordinate ubiquitylation of the nascent protein chain prior to release. Experiments in *Drosophila* and *C. elegans* have suggested that at least in some systems, NMD and non-stop decay can be coupled, and levels of some mRNAs and their associated protein products are regulated by both pathways (Arribere and Fire, 2018; Hashimoto et al., 2017). A forward genetic screen in *C. elegans* further identified the canonical RQC factor PELO (the functional ortholog of dom34/Pelota) as required for repression of an NMD reporter. Based on these and other experiments, the authors proposed a model whereby quality control by NMD in initiated by endonucleolytic cleavage of the mRNA upstream of the stop codon by SMG6. Translation of the resulting truncated mRNA would result in stalling of subsequent ribosomes at its 3′ end, triggering further repression at both the mRNA and protein level by the non-stop decay and RQC pathways (Arribere and Fire, 2018).

If a similar mechanism were occurring in mammalian cells, post-translational degradation of NMD substrates would depend on the canonical RQC factors, including the E3 ubiquitin ligase LTN1, and the ribosome rescue factors pelota and HBS1. However, the majority of RQC factors were not significant hits in either of our screens, although they were identified in an earlier non-stop decay screen performed using matched conditions (Hickey et al., 2020). Furthermore, depletion of LTN1 directly did not affect our NMD reporter under conditions that robustly stabilized a non-stop decay substrate (Fig. 4C). These results suggest that, at least for the class of NMD substrates represented by our reporter, NMD-coupled protein degradation does not rely on the canonical RQC pathway. Together these data suggest a functional separation of nonsense and non-stop decay in mammals, as was observed in *S. cerevisiae* (Arribere and Fire, 2018) and is consistent with the distinct molecular players identified by NMD versus non-stop mRNA decay screens (e.g. Hodgkin et al., 1989; Leeds et al., 1991; Pulak and Anderson, 1993; Wilson et al., 2007).

### Direct ubiquitylation of the nascent NMD polypeptide

The simplest model for NMD-coupled protein degradation is the direct recruitment of an E3 ligase that ubiquitylates the nascent chain while it remains tethered to the ribosome. Earlier studies have suggested that UPF1, a RING domain E3 ubiquitin ligase and core NMD factor that interacts with both the ribosome and eukaryotic release factors, could carry out this role. UPF1 knockdown has been shown to stabilize protein products produced from NMD substrates mRNAs (Kuroha, Tatematsu, and Inada, 2009; Kuroha et al., 2013; Feng, Jagannathan, and Bradley, 2017; Park et al., 2020; Kadlec et al., 2006; Takahashi et al., 2008). Consistent with these reports, UPF1 was identified in our knockdown screen, and depletion of UPF1 stabilized both the mRNA and protein levels of our NMD reporter. However, we found that point mutations to UPF1 that specifically affect its ability to recruit its E2 ubiquitin-conjugating enzyme while leaving its ribosome-binding and helicase domains intact, did not have any effect on the protein-degradation phenotype of our reporter.

We therefore conclude that UPF1 is required for NMD-coupled protein quality control but plays a role that does not depend on its E3 ubiquitin ligase activity. To reconcile these results with previous studies, we propose that UPF1 is involved in the early recognition steps of NMD substrates, which affects both the mRNA and protein degradation branches of NMD. However, our data are inconsistent with a direct role for UPF1 in ubiquitylation of the nascent polypeptide. A dedicated E3 ubiquitin ligase that specifically recognizes nascent chains from NMD substrates was not identified through either the knockdown or knockout genome-wide screens. This is either a limitation of the reporter design, or more likely suggests redundancy between E3s in the recognition event.

### Implications of nascent protein degradation in proteostasis

The identification of a tightly coupled protein degradation branch of NMD has several immediate implications. Most notably, destabilization at the post-translational level will increase the suppression of NMD substrates. Although we find the effects of NMD-coupled protein degradation on our reporters to be modest (∼2-fold), in the context of the cell or an organism, this additional level of regulation might be critical to prevent deleterious or off-target effects. Effects on these fluorescent reporters, which are both overexpressed and in which phenotypes require degradation of the remarkably stable RFP moiety, likely also underestimate the true effect size on an endogenous substrate.

There are numerous physiologically relevant examples where the role of NMD in transcriptome regulation, and subsequent production of potentially aberrant proteins, requires stringent clearance of the nascent product. During histone production, synthesis must be tightly regulated in a manner coupled to the progression of the cell cycle, and the production of even small amounts of downregulated proteins could be problematic. Our results also have implications for viral infection. Co-translational protein degradation is thought to be a key source of peptides for MHC presentation (Balistreri et al., 2014; Fontaine et al., 2018; Wada et al., 2018; Yewdell and Nicchitta, 2006), with viral messages often targeted by NMD (Balistreri et al., 2014; Fontaine et al., 2018; Wada et al., 2018).

Finally, NMD plays an important role in a wide range of genetic diseases – over one third of all human genetic disorders are caused by PTC-creating mutations, including muscular dystrophy and cystic fibrosis (Mort et al., 2008). Although it is generally protective, for numerous disease-causing mutations, the NMD pathway contributes to pathogenesis by suppressing expression of partially functional mutant proteins (∼11% of mutations that cause human disease; Mort et al., 2008). The characterization of a second, parallel branch of NMD and the initial identification of potential factors involved in NMD-coupled protein quality control therefore might represent a valuable platform from which to identify potential targets for the new therapeutic strategies.

## MATERIALS AND METHODS

### Plasmids and antibodies

Reporter constructs for expression in mammalian cells were generated in either the pcDNA5/FRT/TO (Thermo Fisher Scientific) backbone (for expression in HEK293T cells) or the SFFV-tet3G lentiviral backbone with a 3′ WPRE element (for inducible expression in K562 cells; Jost et al., 2017). To create the NMD reporters described in Fig. 1, a fragment of the β-globin gene spanning the last 221 nucleotides of exon 2 (the last 35 nucleotides for inert EJC), intron 2 and 129 nucleotides of exon 3 was amplified via PCR from human genomic DNA as described previously (Pereverzev et al., 2015). Either one or two copies were inserted into the 3′ UTR of a plasmid encoding GFP-P2A-RFP to generate NMD1 and NMD2 respectively. In the lentiviral constructs, the reporters were inserted in reverse orientation to prevent splicing of the introns during lentiviral production. The presence of functional introns was checked via PCR, using primers that should span the introns (Fig. S1B). For this, the RNeasy kit (#74104, Qiagen) was used to purify total RNA from HEK293T transiently expressing the NMD1, NMD2 or the inert EJC reporter. cDNA was obtained by reverse transcription using the SuperScript III First Strand Synthesis SuperMix (#11752, Invitrogen). PCR amplification from this cDNA with respective primers generated a shorter fragment than that of the reporter plasmids, indicating the introns have been spliced out efficiently.

Modifications of the NMD constructs were created by either replacing the P2A site with a glycine-serine linker of identical length for the linked constructs (Fig. 1D), reversing the order of the GFP and RFP for the ‘reverse’ constructs as in Chu et al., 2021, or appending the villin headpiece domain (bVHP) downstream of the RFP (Fig. S2D). For immunoprecipitation experiments, a FLAG tag was appended to the N-terminus of RFP (Fig. 2C). Of note, mEGFP and mCherry versions of the GFP and RFP were used throughout this study, but for simplicity are referred to as GFP and RFP, respectively.

cDNA for UPF1 was acquired from Addgene (#99146) and cloned downstream of a BFP-P2A sequence contained in a lentiviral backbone. This was driven by an EF1α promoter from an upstream ubiquitous chromatin opening element (UCOE). The main isoform of UPF1 (isoform 2) was used, as it has been more comprehensively characterized (Nicholson et al., 2014; Fritz et al., 2022). A mutant of UPF1 with mutations in the RING domain (S134A, N148A and T149A) that disrupts binding with E2 ligases was also acquired from Addgene (#99144). Plasmids containing siRNA-resistant FLAG-tagged SMG6 (wild-type and a D1353A mutant) were a kind gift from Niels Gehring (Institute for Genetics, University of Cologne, Germany).

To generate knockdowns, sgRNAs against LTN1 (5′-GACTCTGAGCACTCAGACCC-3′), CASC3 (5′-GTGCGTAAGTACCTCGCCGG-3′), and DCP1A (5′-GGCGCTGAGTCGAGCTGGGC-3′) were generated by annealed cloning of top and bottom oligonucleotides (Integrated DNA Technologies, Coralville, IA) into a lentiviral pU6-sgRNA EF1α-Puro-T2A-BFP vector digested with BstXI/BlpI (Addgene #84832). BFP was removed when the color interfered with the reporter construct. In certain cases, we used a programmed dual sgRNA guide vector (Addgene #140096) to increase the efficiency of knockdown such as for UPF1 (5′-GGCCGCTCGCAGCCTAGAGC-3′ and 5′-GTTCGAGGGGAGCTGAGGCG-3′) and CNOT4 (5′-GGAGACTCTCAGCTTTCGGT-3′ and 5′-GGGGCCACCATCTTACATTA-3′).

The following antibodies were used in this study: FLAG (#A2220, Sigma, 1:10,000), HA (#A2095, Sigma, 1:1000), UPF1 (#A300-038A, Bethyl, 1:1000), α-tubulin (#T9026, Sigma, 1:5000), CNOT4 (#12564-1-AP, Proteintech, 1:1000), SMG6 (#ab87539, Abcam, 1:1000). Antibodies against GFP and RFP were a kind gift from Ramanujan Hegde (Division of Cell Biology, MRC Laboratory of Molecular Biology, UK). Secondary antibodies used were HRP-conjugated anti-rabbit-IgG (#170-6515, BioRad, 1:5000) and anti-mouse-IgG (#172-1011, BioRad, 1:5000), and HRP-conjugated donkey anti-goat-IgG (ab97110, Abcam, 1:5000).

### siRNAs

Pre-designed Silencer Select siRNAs were ordered from Thermo Fisher Scientific – control (scrambled 1) and SMG6 (s23489).

### Mammalian cell culture

HEK293T cells (ATCC, #CRL-3216) were grown in Dulbecco's modified eagle medium (DMEM; Thermo Fisher Scientific, cat. #21013024) with 10% FBS (Atlanta Biologicals, #S11550) and 2 mM L-glutamine (Invitrogen, #25030081). siRNA treatments were performed according to the manufacturer's instructions in a six-well plate with 30 pmol of each siRNA, allowing knockdown for a total of 72 h. siRNA-treated cells were transiently transfected with 1 μg of reporter construct DNA 24 h prior to harvesting.

Stable HEK293 cell lines were generated using Flp-In 293 T-Rex cells purchased from Thermo Fisher Scientific (USA) (RRID: CVCL_U427). Cell lines were grown in DMEM supplemented with 2 mM glutamine, 10% (w/v) FBS, 15 µg/ml Blasticidine S (Thermo Fisher Scientific, cat. #A1113903; CAS: 3513-03-9) and 100 µg/ml Zeocin (Thermo Fisher Scientific, cat. #R25005). The open-reading frame to be integrated into the genomic FRT site was cloned into the pcDNA5/FRT/TO vector backbone and cell lines were generated according to the manufacturer's protocol. Briefly, the reporter construct was transfected together with pOG44 Flp-In recombinase in a 9:1 ratio using Trans-IT 293 transfection reagent (Mirus, USA) according to the manufacturer's instructions. At 48 h after transfection, 100 µg/ml Hygromycin B (Millipore, cat. #400051-100KU) was used to select for cells that had undergone successful integration.

K562-dCas9-BFP-KRAB Tet-On cells (from the Weissman lab as described in Jost et al., 2017) were grown in RPMI-1640 medium with L-glutamine and HEPES supplemented with 10% Tet System Approved FBS, 100 units/ml penicillin and 100 μg/ml streptomycin (Invitrogen, #15140148). For certain reporter assays, K562 CRISPRi Zim3-hygro Tet-On cells (from the Weissman lab as described in Replogle et al., 2022) were used to promote better knockdown. Cells were maintained at a confluency between 0.5–2×10^6^ cells/ml. All cells were tested for contamination regularly.

### Lentivirus

Lentivirus was produced by co-transfecting HEK293T cells with two packaging plasmids (pCMV-VSV-G and delta8.9, Addgene #8454) and the desired plasmid using TransIT-293 (Mirus) transfection reagent. At 48 h after transfection, the supernatant was collected, centrifuged and flash frozen. In all instances, virus was rapidly thawed prior to transfection. Virus for the genome-wide CRISPRi screen was generated using this method.

### Virus generation for genome wide CRISPR knockout screen

HEK-293T cells were seeded at a density of 750,000 cells/ml in 20 ml viral production medium – IMDM (Thermo Fisher Scientific #1244053) supplemented with 20% inactivated fetal serum (GeminiBio #100-106). After 24 h, the medium was changed to fresh viral production medium. At 32 h post-seeding, cells were transfected with a mix containing 76.8 µl Xtremegene-9 transfection reagent (Sigma Aldrich #06365779001), 3.62 µg pCMV-VSV-G (Addgene #8454), 8.28 µg psPAX2 (Addgene #12260), and 20 µg sgRNA plasmid and Opti-MEM (Thermo Fisher Scientific #11058021) to a final volume of 1 ml. Medium was changed 16 h later to fresh viral production medium. At 48 h after transfection, virus was collected and filtered through a 0.45 µm filter, aliquoted and stored at −80°C until use.

### Generation of K562 reporter cell lines for screening

K562 reporter cell lines were generated by co-transfecting our control or NMD2 viral vectors along with a tet activator element into K562 wild-type or K562-dCas9-BFP-KRAB Tet-On cell lines (from the Weissman lab as in Jost et al., 2017) at one copy number per cell. Positive cells were isolated via FACS on a BD FACSAria2 and grown up to create monoclonal cell lines.

### Flow cytometry analysis

HEK293T cells were analyzed by flow cytometry 24 h after either transient transfection with indicated reporters. T-Rex HEK293 cells stably expressing designated reporters were induced for 24 h prior to harvesting for flow cytometry. For this, cells were first incubated with trypsin before collection (500 ***g*** for 5 min); the cell pellet resuspended in 300 μl of PBS containing 1 μM Sytox Blue Dead Cell Stain (Thermo Fisher Scientific, #S34857) and analyzed on a Miltenyi Biotech MACSQuant VYB Flow Cytometer. For certain experiments, such as treatment with MG132, K562-dCas9-BFP-KRAB Tet-On NMD2 or control monoclonal cell lines (also used for screening) were induced for 24 h with 1 μg/ml doxycycline. For transient reporter experiments, K562 Zim3 or KRAB CRISPRi cells were spinfected at a confluency 0.5×10^6^ cells/ml. Medium was supplemented with 8 μg/ml polybrene (Millipore Sigma, #107689-100G) and the lentivirus of interest was added to the well. The components were mixed by pipetting, and immediately spun down at 1000 ***g*** for 2 h at 30°C. Expression of the reporter constructs was induced with 1 μg/ml doxycycline, and cells were typically analyzed 24 h later unless otherwise indicated. To induce knockdown, cells were spinfected with both guide and reporter, allowed to grow for 8–10 days and then induced with doxycycline. Guide positive cells were selected with 1 µg/l puromycin for 3 days. Flow cytometry data was analyzed either in FlowJo v10.8 Software (BD Life Sciences) or Python using the FlowCytometryTools package.

### qPCR analysis

Relative mRNA levels were determined by quantitative PCR. Total cellular RNA was purified from cells using the RNeasy kit (#74104, Qiagen), treated with DNase I (#18068015, Invitrogen) and reverse transcribed using the SuperScript III First Strand Synthesis SuperMix (#11752, Invitrogen), before being subjected to analysis on a StepOnePlus Real-Time PCR system. The relative expression ratios between sample cDNA levels were then analyzed, using primers that amplified either GFP and RFP, and the housekeeping gene HPRT1 (IDT, Hs.PT.58v.45621572). Each set of primers was checked against a standard dilution curve, and the primer efficiencies were between 90 and 110%. The efficiencies were considered in the expression ratio calculation. The primers used were: GFP (fwd: 5′-ATTGGACGGAGACGTGAATG-3′, rev: 5′-GTTTCCCGGTAGTGCAGATAA-3′) and RFP (fwd: 5′-CCCGCAGACATTCCTGATTA-3′, rev: 5′-AGTCCTGAGTCACTGTAACAAC-3′).

### Inhibition of the ubiquitin-proteasome pathway

To look at the effect of MG132 treatment on the NMD2 reporter as shown in Fig. 2A, wild-type HEK293T cells were transiently transfected with FLAG-tagged versions of the reporter constructs. After 18 h, cells were then treated with either 10 μM of the proteasome inhibitor MG132 (Calbiochem, #474790), or a DMSO control for 6 h. To test the effect of E1 inhibition, this was modified such that cells were treated with either 10 μM of the E1 inhibitor MLN7243 (MedChemExpress, cat. #HY-100487) or DMSO for 8 h. To allow for blotting, cells were then harvested and lysed in 1% SDS. The lysates were normalized to GFP protein levels by serial dilutions and western blotting. The normalized lysates were analyzed by SDS-PAGE and western blotting using anti-FLAG and anti-GFP antibodies. For Fig. 2B, our K562 CRISPRi NMD2 monoclonal cell line was induced with 1 μg/ml doxycycline for 10 h and subsequently treated with 10 μM MG132 or DMSO for 6 h. Cells were harvested and analyzed by flow cytometry on an Attune NxT Flow Cytometer.

To directly observe ubiquitylation of RFP and GFP (Fig. 2C), we generated a stable cell line constitutively expressing HA-tagged ubiquitin conjugated to a BFP marker in HEK293T cells. These cells were transiently transfected with reporters where the RFP was FLAG-tagged and incubated for 42 h. Cells were then treated with 10 μM MG132 for 6 h. For blots, cells were harvested by first being resuspended in lysis buffer [50 mM Hepes pH 7.4, 100 mM KOAc, 2 mM MgAc_2_, 1× cOmplete, EDTA-free protease inhibitor cocktail (Roche, cat. #4693132001), 1 mM DTT, 50 μM PR-619, 10 μg/ml digitonin] and left on ice for 15 min. Mechanical lysis was performed with 10 strokes of a glass dounce and total samples were taken. The amount of RFP and GFP in each sample was determined using a plate reader. Samples for RFP and GFP immunoprecipitations (IPs) were normalized to equivalent RFP and GFP levels respectively, using HA-Ub-containing cell lysate to maintain the total protein concentration. For the RFP IP, SDS was added to 1% final concentration, and the samples were boiled. They were then diluted with IP buffer (50 mM Hepes pH 7.4, 100 mM KOAc, 2 mM MgAc_2_ and 1% Triton X-100) to a final concentration of 0.1% SDS. Samples were immunoprecipitated with anti-FLAG M2 affinity resin (Millipore-Sigma) and eluted with SDS. For the GFP Ips, SDS was added to 1% final concentration, then samples were diluted with IP buffer without boiling. Magnetic beads (Pierce) were coupled to a biontinylated version of a GFP nanobody as described previously (Pleiner et al., 2020), and then were used to immunoprecipitate GFP. Samples were eluted with SDS. The resulting samples were analyzed by western blotting.

### CRISPRi knockdown screen

The genome-scale CRISPRi screen was performed similarly to previously described screens (Gilbert et al., 2014; Horlbeck et al., 2016). The hCRISPRi-v2 compact library (containing 5 sgRNAs per gene, Addgene pooled library #83969) was transduced in duplicate into 330 million K562-dCas9-BFP-KRAB Tet-On-NMD2 cells at a multiplicity of infection (MOI) <1 (the percentage of transduced cells 48 h after infection as determined from the proportion of BFP-positive cells was 20–40%). Cells were grown in 1 l of medium in 1 l spinner flasks (Bellco, SKU: 1965-61010) for the duration of the screen. At 48 h after spinfection, cells were selected with 1 mg/ml puromycin for 3 days. After a 36 h recovery, cells were induced with 1 μg/ml doxycycline for 24 h and sorted on a FACS AriaII Fusion Cell Sorter. The cells were maintained at 0.5×10^6^ cells/ml for the duration of the screen. This ensured that the culture was maintained at an average coverage of more than 1000 cells per sgRNA for the whole screen.

Cells with high BFP (transduced cells) and with both GFP and RFP signal (successfully induced) were gated. Cells were sorted according to the RFP:GFP ratio of this population.

Around 40×10^6^ cells with either the highest (30%) and the lowest (30%) RFP:GFP ratio were collected, pelleted (1000 ***g*** for 20 min) and flash frozen. Genomic DNA was purified using the Nucleospin Blood XL kit (Takara Bio, #740950.10) and amplified with barcoded primers by index PCR. The library (∼264 bp) was purified using SPRIbeads (Bulldog Bio, CNGS005), its concentration measured by Qubit fluorometer (Invitrogen) and its integrity checked by Agilent 2100 Bioanalyzer. Samples were analyzed using an Illumina HiSeq2500 high-throughput sequencer. Sequencing reads were aligned to the CRISPRi v2 library sequences, counted and quantified (Horlbeck et al., 2016). Generation of negative control genes and calculation of phenotype scores and Mann–Whitney *P*-values was performed as described previously (Gilbert et al., 2014; Horlbeck et al., 2016). Gene-level phenotypes and counts are available in Table S1.

### K562 genome-wide CRISPR knockout screen

A genome-wide lentiviral sgRNA library in a Cas9-containing vector (Table S3) was used to transduce 500×10^6^ monoclonal K562 cells containing a tet element and the NMD2 reporter. All other conditions were identical to those used for the CRISPRi KD screen. Cells were induced either at 7, 9 or 11 days with 1 μg/ml doxycycline for 24 h and sorted on a FACS Aria II Fusion cell Sorter on days 8, 10 or 12. Data was processed using the pipeline described above and validated by analysis using MAGeCK (Li et al., 2014). Gene-level phenotypes and counts are available in Table S2.

For extraction of genomic DNA, QIAamp DNA Blood Maxiprep Kit (Qiagen) was used according to manufacturer's instructions with the following modifications: 500 µl of a 10 mg/ml solution of ProteinaseK in water was used in place of QIAGEN Protease; incubation with ProteinaseK and Buffer AL was performed overnight; centrifugation steps after Buffer AW1 and AW2 were performed for 2 min and 5 min, respectively; gDNA was eluted for 5 min using 1 ml of water preheated to 70°C, followed by centrifugation at 400 ***g*** for 5 min. gDNA concentration was determined using the Qubit dsDNA HS Assay kit (Thermo Fisher Scientific #Q32851).

All plasmids and reagents are available upon request.

## Supplementary Material

10.1242/joces.261216_sup1 Supplementary information
