# Supplementary material for: Coupled protein quality control during nonsense-mediated mRNA decay
Source: J Cell Sci. 2023 May 23;136(10):jcs261216. doi: 10.1242/jcs.261216 (PMC10234110; doi:10.1242/jcs.261216)
Supplement: Supplementary information [file joces-136-261216-s1.pdf]

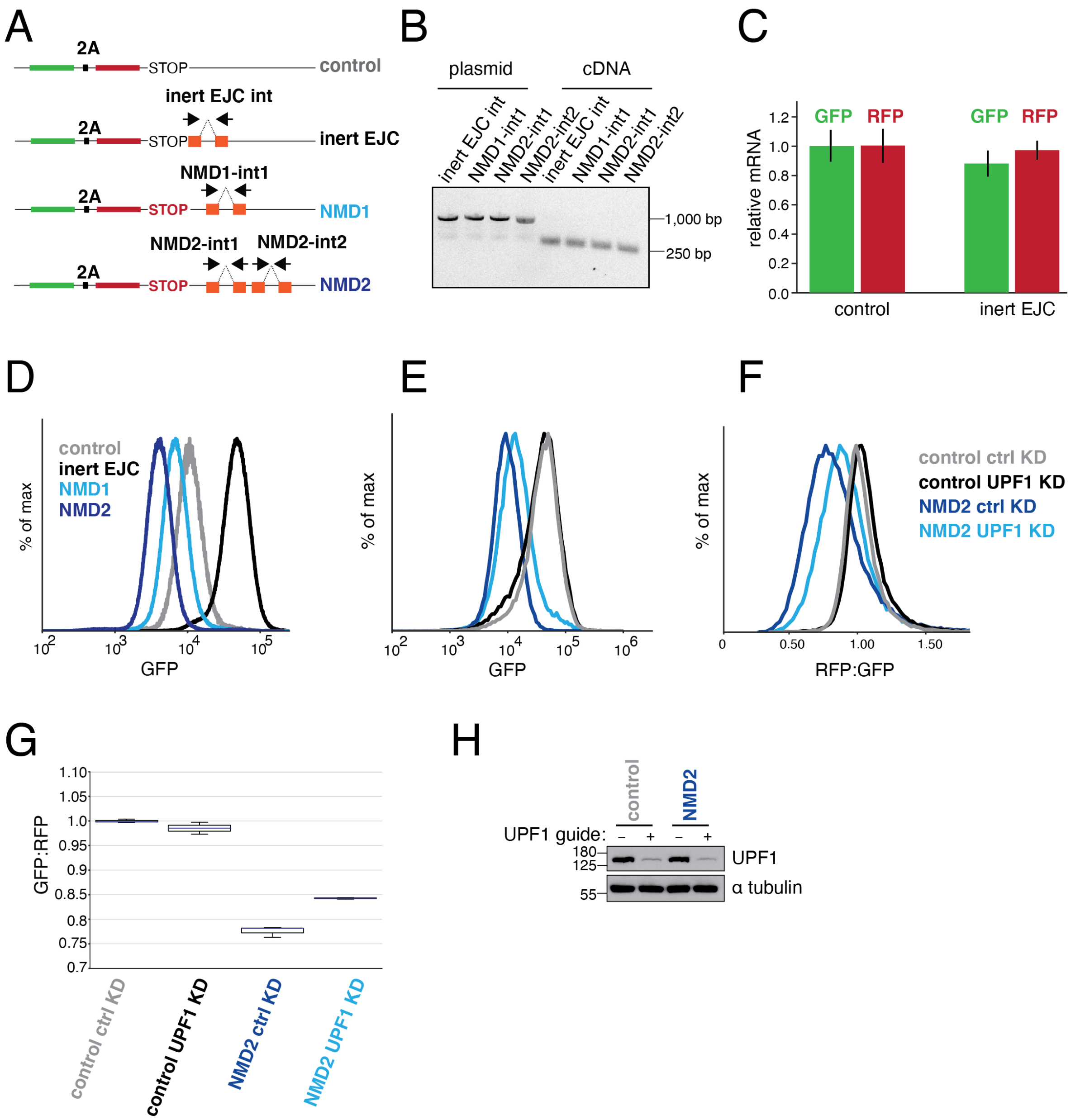

Fig. S1. Characterization of the NMD reporter constructs. (A) Reporter design as depicted and described in Fig. 1A with additional annotation. Orange boxes represent the sequence from the β-globin gene, with the dashed lines representing constitutive splice sites. For NMD1 and NMD2, the first intron was inserted ~200 bp from the stop codon (int1). The NMD2 reporter contained a second β-globin splice site (int2) that resulted in further suppression of the mRNA levels. To account for changes in translation associated with the deposition of an exon junction complex (EJC) following splicing, we created an additional control with the β-globin intron only 12 bp from the stop codon, an insufficient distance for recognition by NMD machinery. Black arrows indicate the position of primers used to check efficient splicing in (B). **(B)** Both β-globin introns were efficiently spliced in their ectopic context. Either plasmid DNA or the corresponding cDNA for the indicated reporters was amplified by PCR using primer pairs that

Journal of Cell Science • Supplementary information

span the introns. Upon splicing, the size of the expected fragment decreases from 1 kb to ~250 bp. **(C)** T-Rex HEK293 cell lines stably expressing either the control or the inert control were induced with doxycycline for 24 hours and the total mRNA was then purified. Relative mRNA levels were determined by RT-qPCR using two sets of primers that anneal to the very 5′ region of the GFP and 3′ region of the RFP open reading frames respectively. The results were normalized to the control and the standard deviation from three independent experiments is displayed. **(D)** GFP channel of samples analyzed by flow cytometry in Fig. 1C. **(E, F)** K562 CRISPRi cells stably expressing the NMD2 reporter or the control reporter under an inducible promoter were infected with guides targeting UPF1 or a non-targeting control. After 8 days of knock-down, reporter expression was induced with doxycycline, and cells harvested after 24 hours for analysis by flow cytometry. The GFP levels are shown in A, and the RFP:GFP ratios are shown in **(G)**, with ratios from three independent experiments quantified on the right. **(H)** Knockdown was validated by Western blotting against UPF1.

Journal of Cell Science • Supplementary information

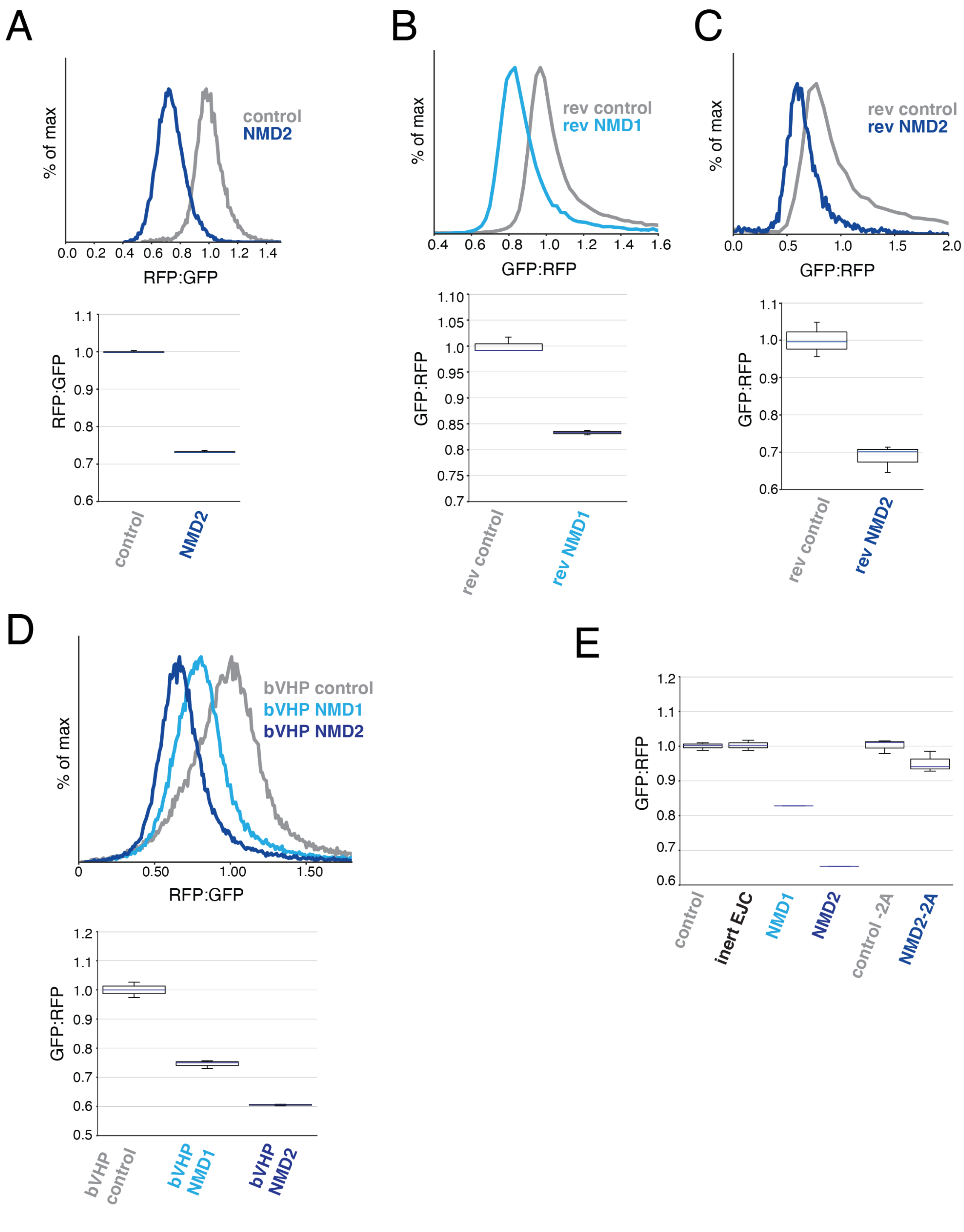

Fig. S2. NMD-linked protein degradation is independent of cell type, fluorescent protein identity and reporter design. (A) K562 CRISPRi cells were virally infected with the control and NMD2 reporters and were then analyzed by flow cytometry after 24 hours of doxycycline induction. Box plots showing the results of three biological replicates are shown below. **(B)** HEK293T cells were transiently transfected with the reversed reporters (in which the GFP and RFP order is reversed), and were analyzed after 24 hours. A box plot showing three biological replicates is below. Note that the NMD1 reverse reporter was used. **(C)** As in A but for the

Journal of Cell Science • Supplementary information

reverse reporters. **(D)** HEK293T cells were transiently transfected with reporters in which a hydrophilic linker domain (bVHP) was inserted between the RFP and the stop codon to ensure the RFP would be fully emerged from the ribosome at the stop codon. The cells were analyzed by flow cytometry after 24 hours, and the results are shown as a histogram. A box plot showing three biological replicates is shown below. **(E)** A box plot showing quantification of three biological replicates for the flow cytometry data shown in Fig. 1D.

Journal of Cell Science • Supplementary information

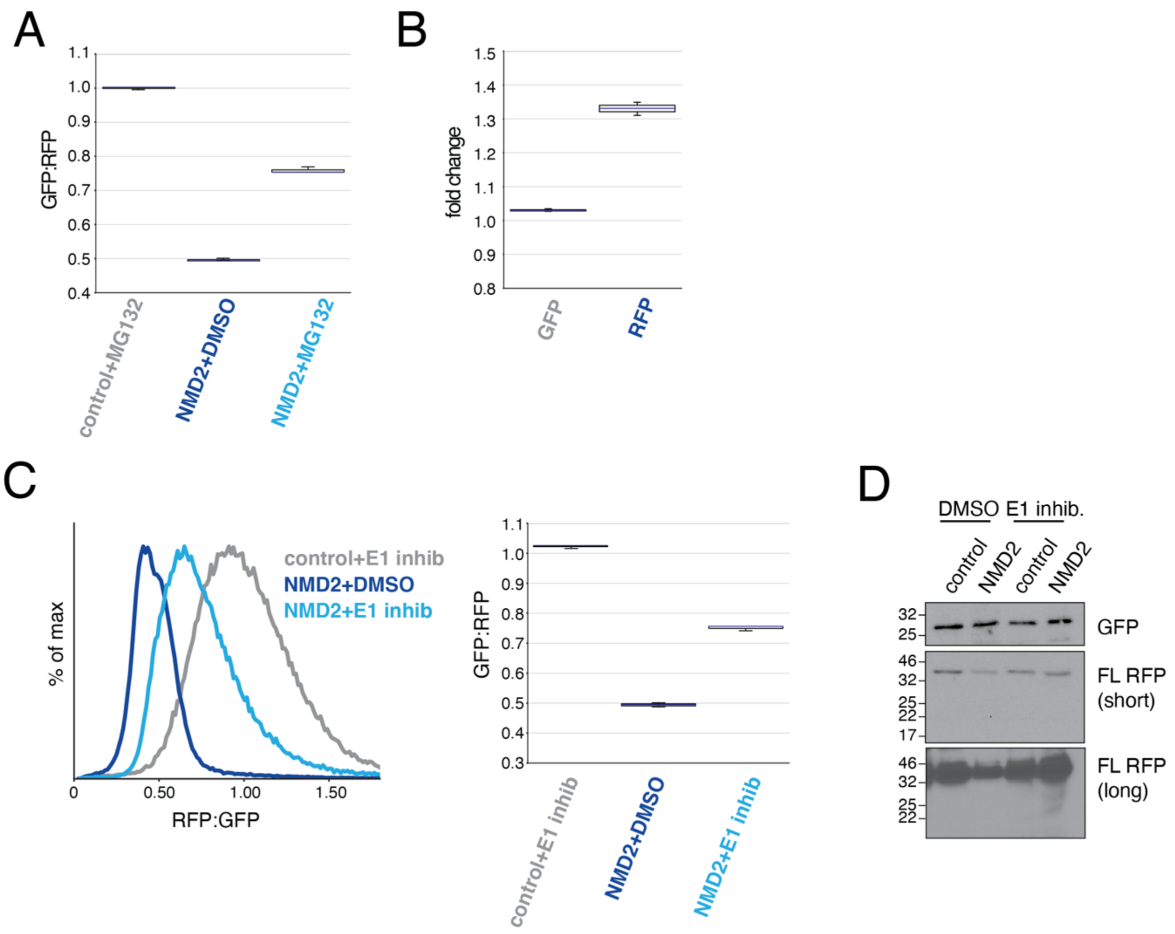

Fig. S3. The ubiquitin-proteasome system mediates NMD-linked nascent protein degradation. (A) A box plot showing three biological replicates for the flow cytometry data presented in Fig. 2A. **(B)** The effect of MG132 treatment on GFP and RFP levels was quantified as fold change for GFP and RFP as shown in Fig. 2B, with three biological replicates plotted. **(C)** HEK293T cells were transiently transfected with either control or NMD2 reporters. After 16 hours, the cells were treated for 8 hours with either 10 µM MLN7243 or a matched DMSO control. Cells were then harvested and analyzed by flow cytometry. The results from three biological replicates are shown on the right. **(D)** Cells were treated as in (C), but were lysed in 1% SDS after MLN7243 treatment. The lysates were boiled and subjected to SDS-PAGE and Western blotting. Samples were normalized to GFP to control for RNA degradation. No RFP degradation products were observed, as seen in the long RFP exposure.

Journal of Cell Science • Supplementary information

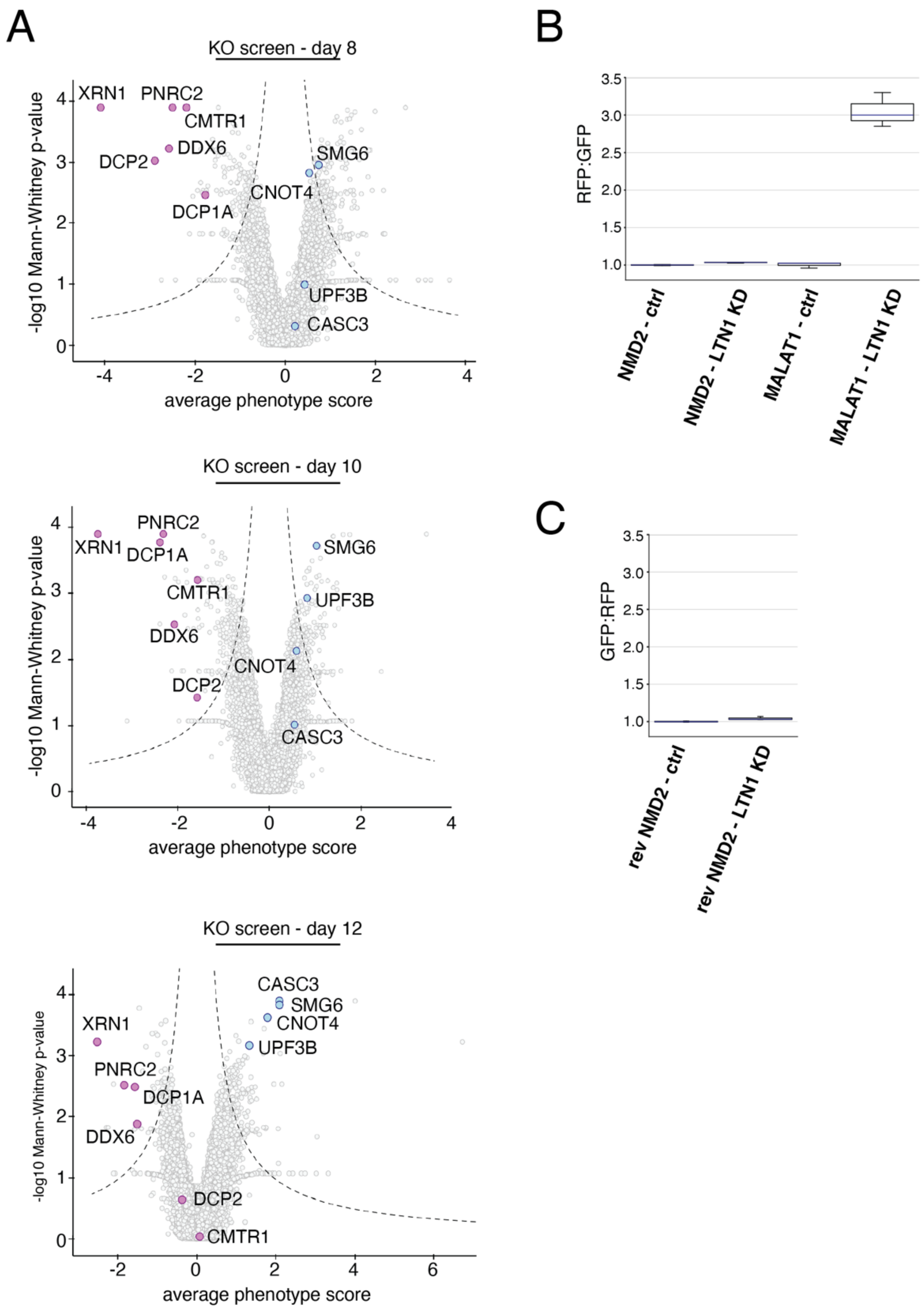

Fig. S4. CRISPR knock-out screen progression across different time points and quantification of NMD dependence on RQC factors. (A) Shown are volcano plots from days 8, 10, and 12 of the knockout CRISPR screen with factors of interest highlighted. Some genes may drop out over the course of the screen, and so show highest phenotype scores at day 8 (e.g. DCP2). Conversely, other genes require a longer time period to be depleted, and show increased effects on the reporter at later time points (e.g. CASC3). The full dataset is available in Supplemental Table 2. **(B)** A box plot showing the results from three biological replicates of the effect of knocking down the RQC E3 ligase LTN1 on the NMD reporter (NMD2) or the non-stop reporter (MALAT1) as shown in Fig. 4C. **(C)** As in **(A)** for the effects of LTN1 knock down on the reverse NMD2 reporter as show in Fig. 4D.

Journal of Cell Science • Supplementary information

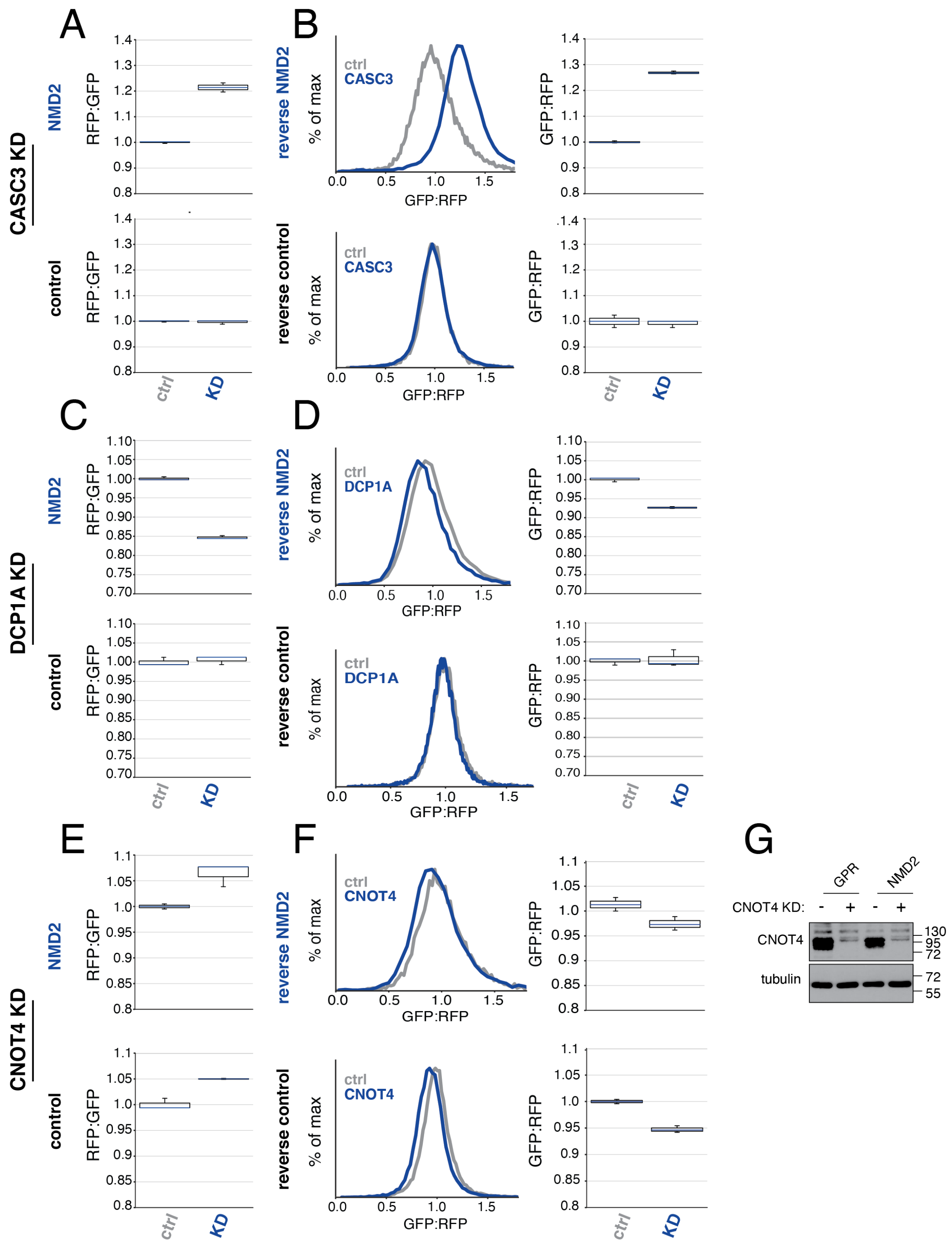

Fig. S5. Validation of the screen hits. (A) Box plots showing the effect of knockdown of CASC3 on the control and NMD2 reporters in K562 Zim3 CRISPRi cells (histograms shown in Fig. 5) across three biological replicates. (B) CASC3 was CNOT4 were depleted by sgRNA for 8 days in K562 Zim3 CRISPRi cells expressing either the reverse NMD2 reporter or the reverse

Journal of Cell Science • Supplementary information

control reporter. Displayed are the RFP:GFP ratios for reporters as determined by flow cytometry after 24 hours of induction. Box plots showing three biological replicates are shown next to each histogram. **(C, D)** As above for DCP1A. **(E, F)** As above for CNOT4. **(G)** CNOT4 depletion was confirmed by Western blot.

Journal of Cell Science • Supplementary information

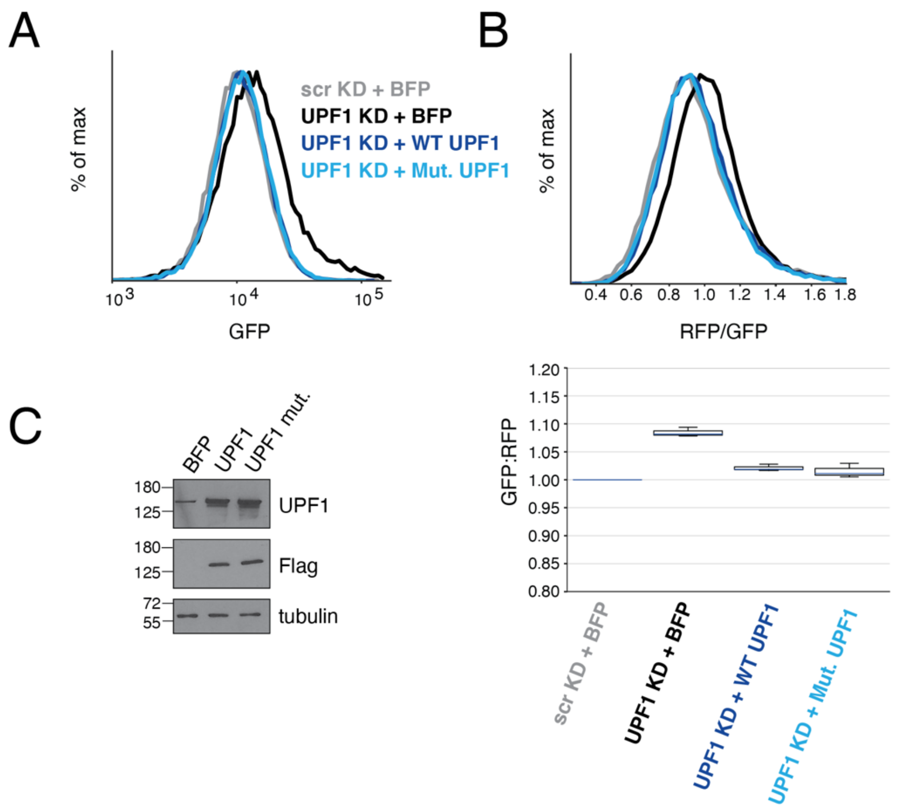

Fig. S6. The role of UPF1's E3 ligase activity in NMD-linked protein degradation. (A) K562 CRISPRi cells stably expressing the inducible NMD2 reporter were constructed to stably express one copy of either BFP, a FLAG-conjugated wild-type UPF1, or a FLAG-conjugated mutant UPF1 (S134A, N148A, T149A) with disruptions that abolish association with E2 conjugating enzymes. WT or mutant UPF1 was separated from BFP by a viral P2A sequence, allowing us to use BFP as a proxy for UPF1 infection. These cells were then infected with dual sgRNA guides targeting UPF1 or a non-targeting control. Note that rescue constructs were resistant to the sgRNA. After 8 days of knockdown, the NMD2 reporter was induced with doxycycline for 24 hours, after which cells were harvested and analyzed by flow cytometry. GFP levels are shown in (**A)** and the RFP:GFP ratios are shown in (**B**), with the quantification of three biological replicates below. (**C**) UPF1 wild-type and mutant over-expression levels were confirmed by Western blotting in the K562 line stably expressing NMD2.

Journal of Cell Science • Supplementary information

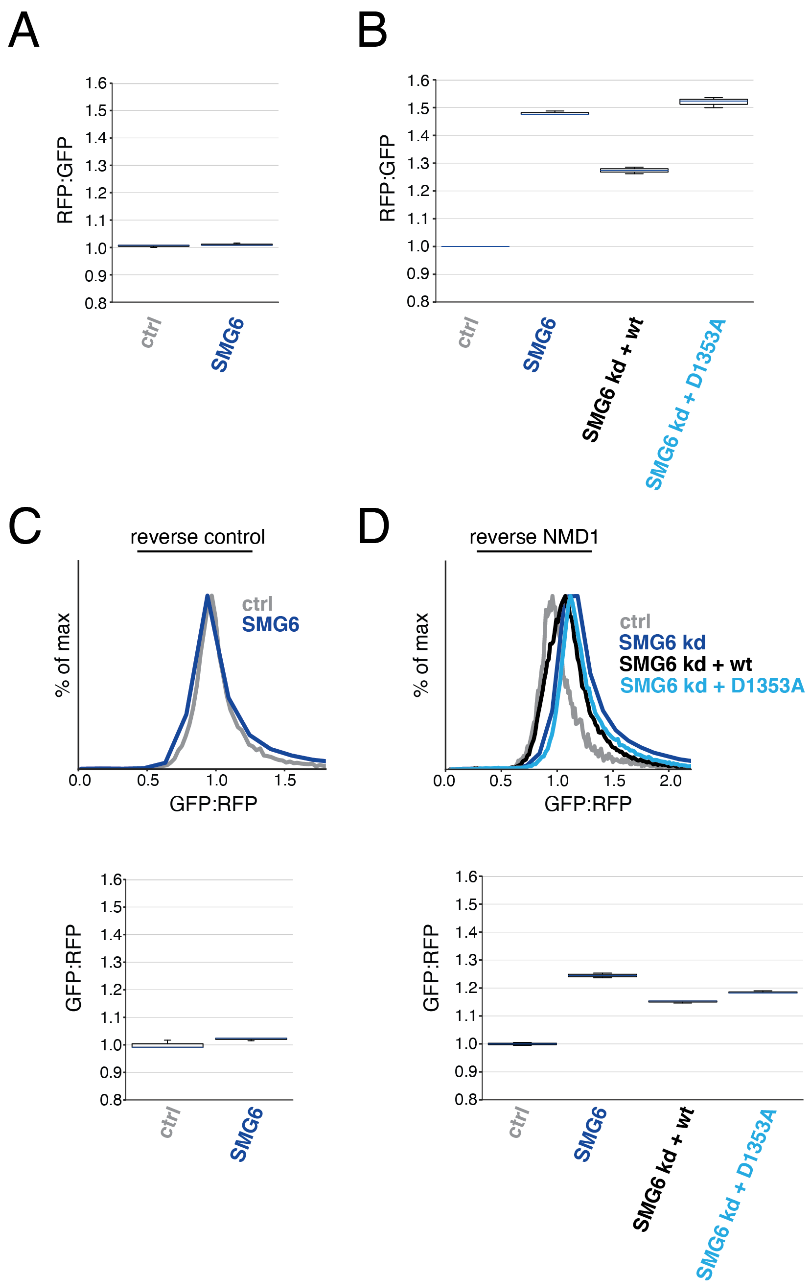

Fig. S7. The effect of SMG6 is independent of fluorescent protein order. (**A**) Box plots showing the effect of SMG6 depletion on the control reporter (Fig. 6A) across three biological replicates. (**B**) As in (A) for effects of SMG6 depletion on NMD2 with rescue by the wild type and mutant SMG6. **(C)** HEK293T cells were treated with siRNA against SMG6 for 48 hours, then were transiently transfected with the reversed control reporter. Cells were analyzed by flow cytometry after 24 hours, and the experiment was performed in triplicate (results plotted below). **(D)** HEK293T cells were treated with an siRNA against SMG6 as in (C), then were transfected with an siRNA-resistant version of either wild-type SMG6 or a PIN domain mutant version and the reversed NMD reporter. The cells were analyzed by flow cytometry after 24 hours. A box plot showing the results from three biological replicates is shown.

Journal of Cell Science • Supplementary information

Table S1. Genome-wide CRISPRi NMD screen

Click here to download Table S1

Table S2. Genome-wide CRISPR knock-out NMD screen

Click here to download Table S2

Table S3. CRISPR knock-out library sgRNA list

Click here to download Table S3

Journal of Cell Science • Supplementary information
